# Application of a Photocatalyst as an Inactivator of Bovine Coronavirus

**DOI:** 10.3390/v12121372

**Published:** 2020-11-30

**Authors:** Nobuki Yoshizawa, Ryoko Ishihara, Daisuke Omiya, Midori Ishitsuka, Shouichirou Hirano, Tohru Suzuki

**Affiliations:** 1 Division of Pathology and Pathophysiology, Hokkaido Research Station, National Institute of Animal Health, NARO, Sapporo, Hokkaido 062-0045, Japan; yoshizawan334@affrc.go.jp (N.Y.); rishihara@affrc.go.jp (R.I.); 2 Ehime Prefectural Livestock Disease Diagnostic Center, Toon, Ehime 791-0212, Japan; 3 Bio and Healthcare Business Division, Tsukuba Technical Center, Wako Filter Technology Co., Ltd., Bando, Ibaraki 306-0616, Japan; daisuke.oomiya@waftec.jp (D.O.); midori.ishituka@waftec.jp (M.I.); shouichirou.hirano@waftec.jp (S.H.)

**Keywords:** bovine coronavirus, photocatalyst, antiviral activity, visible light irradiation, inactivator

## Abstract

Bovine coronavirus (BCoV), a major causative pathogen of bovine enteric and respiratory diseases and a zoonotic pathogen transmissible between animals and humans, has led to severe economic losses in numerous countries. BCoV belongs to the genus *Betacoronavirus*, which is a model of a pathogen that is threatening human health and includes severe acute respiratory syndrome coronavirus (SARS-CoV), SARS-CoV-2, and Middle East respiratory syndrome coronavirus. This study aimed to determine whether photocatalytic material effectively reduces CoVs in the environment. Using the film adhesion method of photocatalytic materials, we assessed its antiviral activity and the effect of visible light irradiation according to methods defined by the International Organization for Standardization. Consequently, photocatalytic material was found to have antiviral activity, reducing the viral loads by 2.7 log TCID_50_ (tissue culture infective dose 50)/0.1 mL (500 lux), 2.8 log TCID_50_/0.1 mL (1000 lux), and 2.4 log TCID_50_/0.1 mL (3000 lux). Hence, this photocatalytic material might be applicable not only to reducing CoVs in the cattle breeding environment but also perhaps in other indoor spaces, such as offices and hospital rooms. To our knowledge, this study is the first to evaluate the antiviral activity of a photocatalytic material against CoV.

## 1. Introduction

Coronaviruses (CoVs) belong to the order *Nidovirales*, family *Coronaviridae*, subfamily *Orthocoronavirinae*, and are positive-sense, single-stranded, enveloped RNA viruses with the largest genome of approximately 26–32 kb among the currently known RNA viruses [1]. The subfamily *Orthocoronavirinae* comprises four genera: *Alphacoronavirus*, *Betacoronavirus*, *Gammacoronavirus*, and *Deltacoronavirus* [2,3,4]. CoVs can be detected from various animal species, including cattle, pigs, camels, mice, dogs, cats, bats, birds, and humans, and cause various symptoms in the respiratory, hepatic, and gastrointestinal tracts and mild-to-severe neurological disorders [5,6,7,8]. *Gamma-* and *Deltacoronaviruses* generally infect birds; however, some of them can also infect mammals [3]. On the other hand, *Alpha-* and *Betacoronaviruses* infect only mammals and usually cause respiratory illness in humans [9,10].

The genus *Betacoronavirus* further comprises four lineages (A–D): (A) *Embecovirus*, including mouse hepatitis virus (MHV), bovine coronavirus (BCoV), equine coronavirus (ECoV), and human coronaviruses (HCoV-OC43 and HCoV-HKU1); (B) *Sarbecovirus*, including severe acute respiratory syndrome coronavirus (SARS-CoV) and SARS-CoV-2; (C) *Merbecovirus*, including Middle East respiratory syndrome coronavirus (MERS-CoV); and (D) *Nobecovirus*, including several bat coronaviruses [11]. Most *Betacoronaviruses* are commonly found in bats, and numerous diverse CoVs phylogenetically related to SARS-CoV and MERS-CoV have been reported in various bat species worldwide [4]. Therefore, HCoVs other than HCoV-OC43 seem to have originated from bats.

In contrast, HCoV-OC43 may result from interspecies transmission of BCoV to humans [12]. Furthermore, BCoV-like viruses have been isolated from various wild ruminants and a child with acute diarrhea [13,14,15,16]. Therefore, BCoV seems to be a zoonotic pathogen transmissible between animals and humans, resulting in public health concerns. BCoVs associate with bovine enteric disease (BED), such as neonatal calf diarrhea and winter dysentery in adult cattle, and also associate with bovine respiratory disease (BRD) in cattle of all age groups [17]. In Japan, BCoV has been most frequently detected in cattle with diarrhea [18,19]. Furthermore, BCoV is the second-most detected pathogen in cattle with pneumonia in Japan [19]. Hence, BCoV infections reduce milk production, deteriorate health, and lead to the death of calves, resulting in severe economic losses. However, there is no effective method to prevent BCoV infections, except for infection control and hygiene management.

CoVs are primarily transmitted through the inhalation of excretions from infected individuals and through direct contact with contaminated surfaces followed by touching the nose, mouth, and eyes [20,21]. These viruses reportedly stabilize in favorable atmospheric conditions on different surfaces for days [22]. In addition, HCoV has been reported on the surface of door handles, cell phones, and other items in the houses of confirmed patients [23]. Moreover, transmission in an unventilated environment or closed spaces owing to high aerosol concentrations has been previously suggested [24]. Furthermore, fecal–oral transmission is also an important route because the viruses are shed in the feces for a long period [25].

There are several preventive treatment methods for CoV infections, such as antiviral drugs, vaccines, traditional medicines, passive immunization, and inactivation agents [26,27,28,29]. Furthermore, the agents for inactivating and disinfecting plural viruses include spraying antiseptic solution such as ethanol, applying acidic or alkaline solutions, ultraviolet (UV) irradiation, heat treatment, and the use of material with antiviral activity [30,31,32]. Among these, the use of photocatalytic material is a more suitable method because it requires only light energy from solar and fluorescent irradiation. Photocatalysts with visible light irradiation can inactivate several viruses, including avian influenza virus A (H1N1) and human adenovirus, which can threaten human health [33,34,35]. However, no studies have assessed the efficacy of photocatalysts against CoVs.

This study aimed to investigate the antiviral activity against BCoV with visible light irradiation as a model to prevent a potential CoV infection. Consequently, the photocatalytic material used in this study displayed antiviral activity and the effect of visible light irradiation against BCoV. This material is potentially applicable as an effective tool to reduce CoVs in animal and human living and community spaces.

## 2. Materials and Methods

### 2.1. Virus and Cell Culture

The Hokkaido/9/03 strain, originally isolated from nasal swab samples of cattle with pneumonia at a farm in Hokkaido prefecture, Japan, was used in this study [36]. 

The Hokkaido/9/03 strain was inoculated in 75 cm^2^ flasks (10^6^ cells) of HRT-18G cells (ATCC: CRL-11663) and incubated at 37 °C for 1 h in a humidified 5% CO_2_ incubator. Thereafter, cells were washed twice with 20 mL of sterilized phosphate buffered saline (PBS), added to 20 mL of Dulbecco’s modified Eagle’s medium (DMEM) (Nissui, Tokyo, Japan) without fetal bovine serum (FBS), and incubated at 37 °C for 3 days. Thereafter, cells were harvested and centrifuged at 1200× *g* for 5 min to eliminate debris. The virus titer of the supernatant (tissue culture infective dose 50 (TCID_50_)_/_0.1 mL) was determined using the method of Reed and Muench [37] and stored at −80 °C until use. Although the virus titer of the stored Hokkaido/9/03 strain approached 10^7.0^ TCID_50_/0.1 mL, the stored viruses were diluted in half with PBS to prepare a virus suspension to be used in subsequent experiments.

### 2.2. Film Adhesion of Photocatalytic Material and Test Equipment

Photocatalytic material was manufactured by Wako Filter Technology Co., Ltd. (Ibaraki, Japan). The photocatalytic material was composed of a peroxotitanium acid solution (70%) and a peroxo-modified anatase solution (30%), which is responsive to visible light.

The antiviral activity of this photocatalytic material was assessed using the film adhesion method. A transparency film (overhead projector film VF-1410N, KOKUYO Co., Ltd., Osaka, Japan) was cut into 50 × 50 mm^2^ squares and sprayed with the photocatalytic material with a spray gun (LPH-50-062G, ANEST IWATA Corporation, Yokohama, Japan). Thereafter, the film surface was dried with hot air at 60–70 °C. Spraying and drying were repeated alternately to adjust to a 0.2 mg/m^2^ coating weight. A transparency film uncoated with photocatalytic material was used as a negative control.

**Figure 1:**
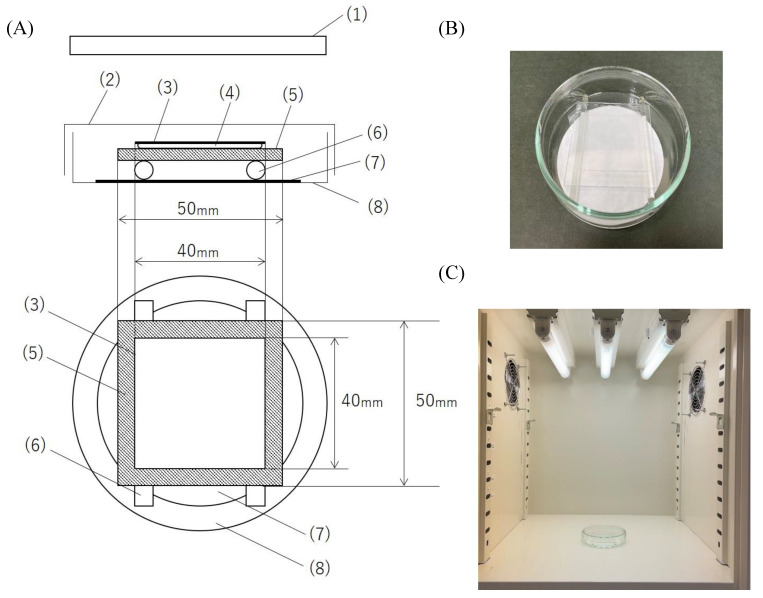
(**A**) Schematic of the test equipment. The test equipment was prepared in accordance with ISO 18071, a guideline of the International Organization for Standardization: (1) light source (fluorescent lamp), (2) Petri dish (lid), (3) cover film, (4) virus suspension, (5) test film, (6) glass rod, (7) paper filter, and (8) Petri dish (bottom). (**B**) A photo of a moisture chamber with a test film. (**C**) A photo of the testing equipment.

The testing equipment was set up in accordance with ISO 18071, a guideline of the International Organization for Standardization [38], facilitating the assessment of the antiviral activity of photocatalytic material under an indoor lighting environment. The equipment comprised a light source and a moisture chamber with a test film (Figure 1).

### 2.3. Assessment of Antiviral Activity of Photocatalytic Material with Visible Light Irradiation

A sterilized moist paper filter was placed at the bottom of a sterilized Petri dish containing 2 mL of sterilized water. Two glass rods were intercalated to avoid contact between the test film and the paper filter. The test film, which was UV irradiated with an irradiance of 1 mW/cm^2^ (380 nm) for 24 h with a black light (06947604, NEC Corporation, Tokyo, Japan) to inactivate organic matter before the test, was placed on them with the photocatalytic-coated surface. In total, 150 μL of the virus suspension (10^6.7^ TCID_50_/0.1 mL) was harvested with a sterilized pipette tip and dropped onto the test film. A 40 × 40 mm^2^ square of sterilized film was placed on top of the dripped suspension and lightly pushed into the suspension to spread it throughout the entire film surface. Thereafter, the lid of the Petri dish was put in place to conserve moisture. The Petri dishes containing the six test films (three photocatalytic-coated films and three photocatalytically uncoated films) with the virus suspension were irradiated with 500, 1000, and 3000 lux visible light with a white fluorescent lamp (600113, Mitsubishi Electric Lighting Corporation, Kanagawa, Japan) for 4 h. The Petri dishes containing the six test films (three photocatalytic-coated films and three photocatalytically uncoated films) with the virus suspension were maintained in the dark for the same duration at 25 ± 3 °C.

After 4 h with or without visible light irradiation, both test films and cover films were washed with 5 mL of DMEM without FBS in a Stomacher bag, and these washout solutions were used to determine the virus titers.

### 2.4. Determination of TCID_50_ of Virus Suspensions on Test Films after 4 h with or without Visible Light Irradiation

Virus titers in suspensions on test films after 4 h with or without visible light irradiation were determined using the TCID_50_ assay on confluent monolayers of HRT-18G cells in 96-well plates (Techno Plastic Products AG, Trasadingen, Switzerland). Cell monolayers were prepared by adding 100 μL of 10^4.2^ cells/mL in DMEM with 5% FBS to each well, and then plates were incubated at 37 °C for 24 h in a humidified 5% CO_2_ incubator. Each collected solution was serially 10-fold diluted in DMEM without FBS. After 100 μL of each dilution was inoculated with confluent cells in 5 wells, the mixture was incubated at 37 °C for 7 days. On day 7 postinoculation, the TCID_50_/0.1 mL of each solution was determined using the aforementioned method [37].

### 2.5. Criteria for Assessment of Antiviral Activity of Photocatalyst and Effect of Visible Light Irradiation

The values of antiviral activity and the effects of visible light irradiation were determined in accordance with ISO 18071 [38] as follows:

Value of antiviral activity of photocatalyst with visible light irradiation:

(V_L_) = log (BL/SL) (1)

Value of antiviral activity of photocatalyst without visible light irradiation:

(V_D_) = log (BD/SD) (2)

Effect of visible light irradiation:

(_Δ_V) = V_L_ − V_D_ (3)

where BL—means of the virus titers of three photocatalytically uncoated films after visible light irradiation with a constant illuminance; SL—means of the virus titers of three photocatalytic-coated films after visible light irradiation with a constant illuminance; BD—means of the virus titers of three photocatalytically uncoated films after being kept in the dark for the same duration as visible light irradiation; SD—means of the virus titers of three photocatalytic-coated films after being kept in the dark for the same duration as visible light irradiation.

The performance of the photocatalytic material was determined to be effective when the antiviral activity of the photocatalyst with visible light irradiation (V_L_) was ≥2.0 and the effect of visible light irradiation (_Δ_V) was ≥0.3, based on the criteria of the Photocatalysis Industry Association of Japan (PIAJ) [39]. V_L_ of 2.0 and _Δ_V of 0.3 correspond with a mean reduction of 1/100 and 1/2 of viruses with visible light irradiation, respectively.

### 2.6. Immunofluorescence Assay

To visualize the antiviral activity of the photocatalytic material irradiated with 1000 lux visible light for 4 h, an immunofluorescence assay (IFA) in addition to the TCID_50_ assay was performed using virus suspensions harvested every 1 h (from 0 to 4 h) after visible light irradiation. Cell monolayers were prepared by adding 300 μL of 10^4.1^ cells/mL in DMEM with 5% FBS per well in an 8-well chamber slide (Thermo Fisher Scientific, Waltham, MA, USA) and incubated at 37 °C for 24 h in a humidified 5% CO_2_ incubator. The harvested solutions were inoculated into confluent cells and then maintained at 37 °C for 1 h. Thereafter, the solutions were replaced with DMEM without FBS. Approximately 22 h later, cells were washed with PBS, fixed with acetone, and incubated with anti-BCoV rabbit serum (originally generated in our laboratory) for 1 h. Subsequently, cells were washed thrice with PBS and incubated with goat FITC (fluorescein isothiocyanate) conjugate anti-rabbit IgA, IgG, and IgM (Cappel Laboratories, Cochranville, PA, USA) for 1 h. Thereafter, cells were washed thrice with PBS and incubated with DAPI (4,6-diamidino-2-phenylindole) solution (Dojindo Laboratories, Kumamoto, Japan) for 10 min. After three washes with PBS, the coverslips were sealed with Mountant Perma Fluor (Thermo Fisher Scientific, Waltham, MA, USA) and observed using a fluorescence microscope (Axiovert200/40 LD-1; Carl Zeiss AG, Oberkochen, Germany).

## 3. Results

### 3.1. Determination of TCID_50_ of Virus Suspensions on Test Films after 4 h with or without Visible Light Irradiation

Virus titers in virus suspensions on test films uncoated or coated with photocatalytic material harvested after 4 h with or without visible light irradiation of 500, 1000, and 3000 lux are summarized in Table 1.

The mean virus titers from three photocatalytically uncoated films obtained at 0 h were 5.2 log TCID_50_/0.1 mL (500 lux), 4.3 log TCID_50_/0.1 mL (1000 lux), and 4.2 log TCID_50_/0.1 mL (3000 lux), respectively. The mean virus titers from photocatalytically uncoated films harvested after 4 h without visible light irradiation displayed no clear differences from those of photocatalytically uncoated films harvested at 0 h. Furthermore, the mean virus titers from three photocatalytic-coated films harvested after 4 h without visible light irradiation exhibited decreases of 2.9 log TCID_50_/0.1 mL (500 lux), 1.6 log TCID_50_/0.1 mL (1000 lux), and 1.8 log TCID_50_/0.1 mL (3000 lux) compared with those from photocatalytically uncoated films harvested at 0 h, respectively. Moreover, the virus titers from three photocatalytic-coated films harvested after 4 h with visible light irradiation showed decreases of 0.4 log TCID_50_/0.1 mL (500 lux), 1.1 log TCID_50_/0.1 mL (1000 lux), and 1.2 log TCID_50_/0.1 mL (3000 lux) compared with those from photocatalytic-coated films harvested after 4 h without visible light irradiation. The values of antiviral activity of photocatalysts with visible light irradiation (V_L_) and the effects of visible light irradiation (_Δ_V) were 2.7 (500 lux), 2.8 (1000 lux), and 2.4 (3000 lux), and 0.5 (500 lux), 0.8 (1000 lux), and 0.6 (3000 lux), respectively, all of which exceeded the criteria (V_L_: 2.0 and _Δ_V: 0.3) of the PIAJ [39].

### 3.2. Immunofluorescence Assay

The virus titers of virus suspensions from photocatalytic uncoated and coated films harvested every 1 h with or without 1000 lux visible light irradiation for 4 h are summarized in Table 2. The mean virus titers from three photocatalytically uncoated films harvested at 0 h were 4.7 log TCID_50_/0.1 mL. Furthermore, the mean virus titers from three photocatalytic-coated films harvested every 1 h with visible light irradiation for 4 h showed decreases of 1.1 log TCID_50_/0.1 mL (1 h), 1.5 log TCID_50_/0.1 mL (2 h), 2.3 log TCID_50_/0.1 mL (3 h), and 2.4 log TCID_50_/0.1 mL (4 h) compared with those of photocatalytically uncoated films harvested at 0 h. Moreover, the values of antiviral activity of photocatalysts with visible light irradiation (V_L_) and the effects of visible light irradiation (_Δ_V) every 1 h were 0.9 (1 h), 1.7 (2 h), 2.0 (3 h), and 2.2 (4 h), and 0.2 (1 h), 0.6 (2 h), 0.8 (3 h), and 0.7 (4 h), respectively, two points of times (3 and 4 h) of which exceeded the criteria (V_L_: 2.0 and _Δ_V: 0.3) of the PIAJ [39].

**Figure 2:**
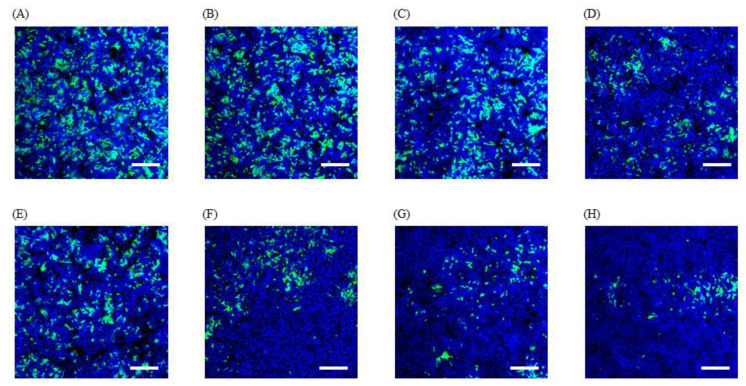
Images of immunofluorescence assay (IFA) using virus solutions from photocatalytic uncoated (**A**–**C**) and coated (**D**–**H**) films harvested every 1 h with or without 1000 lux light irradiation for 4 h. Twenty-two hours after inoculation with the collected virus solutions, cells were stained with anti-BCoV (bovine coronavirus) rabbit serum followed by goat FITC (fluorescein isothiocyanate) conjugate anti-rabbit IgG, IgA, and IgM (green), and with DAPI (4,6-diamidino-2-phenylindole) (blue). (**A**) photocatalytically uncoated films after 0 h without visible light of 1000 lux, (**B**) photocatalytically uncoated films after 4 h without visible light of 1000 lux, (**C**) photocatalytically uncoated films after 4 h with visible light of 1000 lux, (**D**) photocatalytic-coated films after 4 h without visible light of 1000 lux, (**E**–**H**) photocatalytic-coated films after 1 h (**E**), 2 h (**F**), 3 h (**G**), and 4 h (**H**) with visible light of 1000 lux. Scale bar represents 100 μm.

IFA images using virus suspensions from photocatalytically uncoated films revealed no differences in the levels of green fluorescence, regardless of visible light irradiation and the duration of irradiation (Figure 2). The IFA image using virus suspensions from photocatalytic-coated films after 4 h in the dark further decreased the levels of green fluorescence compared with those of the photocatalytically uncoated film after 4 h in the dark. Furthermore, the levels of green fluorescence markedly decreased in IFA images using virus suspensions from photocatalytic-coated films, consistent with the effects of visible light irradiation, compared with those from photocatalytic-coated films in the dark. These results are almost identical to those of the TCID_50_ assay.

## 4. Discussion

The values of antiviral activity of photocatalysts with visible light irradiation (V_L_) using virus suspensions on test films after visible light irradiation for 4 h at 500, 1000, and 3000 lux yielded 2.4–2.8 log TCID_50_/0.1 mL. Furthermore, the effects of visible light irradiation (_Δ_V) at the three values of illumination yielded 0.5–0.8 log TCID_50_/0.1 mL. Both values of antiviral activity of photocatalysts with visible light irradiation (V_L_) and the effects of visible light irradiation (_Δ_V) at all three illuminations exceeded the criteria defined by the PIAJ [39]. Hence, the photocatalytic material used in this study appears to have high antiviral activity against BCoV.

Habibi-Yangjeh et al. reported three mechanisms of viral inactivation by a photocatalyst consisting of a peroxotitanium complex as follows: physical damage of viruses, metal ion toxicity obtained from metal-including photocatalysts, and chemical oxidation by reactive oxygen species generated over the photocatalysts [35]. Moreover, a previous study demonstrated that the adenovirus was destroyed by a photocatalyst under transmission electron microscopy observation [40]. Therefore, the reduction of BCoV loads presented herein might have been caused by these three mechanisms because the photocatalyst used in this study was made from a peroxotitanium complex.

Furthermore, this study shows that the photocatalytic material used here was completely effective under visible light irradiation of 500 lux. The illuminance of solar light is usually >90,000 lux during the daytime [41]; hence, this photocatalytic material can be fully useful outdoors. On the other hand, the illuminance in indoor environments, including offices, hospital rooms, laboratories, and libraries, is generally recommended to be 500 lux in accordance with ISO 8995 [42]; hence, this photocatalyst is potentially useful also under indoor lighting environments. Therefore, this material is potentially applicable as a viral inactivator to reduce not only viral pathogens in environments where cattle are maintained but also the risk of CoV contact infections by spraying various surfaces including walls, floors, knobs, chairs, and handrails inside human living and community spaces.

We observed a reduction in the virus titer on photocatalytic-coated films after 3 h with 1000 lux visible light irradiation based on the criteria defined by the PIAJ [39]. Furthermore, IFA revealed a decrease in the levels of green fluorescence with a reduction in the virus titer. These results indicate that the photocatalytic material used in this study displayed antiviral activity for at least 3 h with visible light irradiation. In a previous study, the load of avian influenza virus (H1N1) on glass coated with platinum-loaded tungsten oxide (Pt-WO_3_) as a photocatalytic material was clearly decreased with 1000 lux visible light irradiation for 2 h [33]. These facts might support photocatalytic materials as a useful tool for viral inactivation to prevent the transmission of enveloped RNA viruses associated with respiratory disorders.

In conclusion, this study shows that the photocatalytic material used here exhibits high performance with visible light irradiation of different illuminances for 4 h. Especially, it is valuable that this material has effective antiviral activity against BCoV with visible light irradiation of 500 lux, an illuminance recommended in indoor environments. These results suggest the possibility of this photocatalyst having antiviral activity against SARS-CoV-2 because BCoV belongs to the same genus as that of SARS-CoV-2, which has propagated and transmitted worldwide. Although further studies are required to analyze the antiviral activity of this material against SARS-CoV-2, this material might be of interest, when properly used, to prevent and reduce CoV infection indoors; however, a possible increase in the concentration of some volatile organic compounds due to photocatalysis may be a concern, as reported in [43].

## Figures and Tables

**Table 1:** Inactivation of bovine coronavirus on photocatalytic uncoated or coated films after 4 h with or without visible light irradiation at different illuminance values.

Time of Irradiation (h)	Illuminance Values of Visible Light Irradiation (lux)	Virus Titers after Visible Light Irradiation ^a^		Antiviral Activity Values ^c^		
		Uncoated ^b^	Coated ^b^	V_L_	V_D_	_Δ_V
0	0	5.2 (±0.1)	N.T.			
4	0	4.5 (±0.2)	2.3 (±0.2)		2.2	0.5
4	500	4.6 (±0.3)	1.9 (±0.4)	2.7		
0	0	4.3 (±0.0)	N.T.			
4	0	4.7 (±0.2)	2.7 (±0.2)		2.0	0.8
4	1000	4.4 (±0.3)	1.6 (±0.1)	2.8		
0	0	4.2 (±0.2)	N.T.			
4	0	4.2 (±0.1)	2.4 (±0.2)		1.8	0.6
4	3000	3.6 (±0.2)	1.2 (±0.5)	2.4		

N.T.: not tested. ^a^ Virus titer is shown in log TCID_50_ (tissue culture infective dose 50)/0.1 mL. The virus titer represents the mean (±standard deviation) of three independent experiments. ^b^ Uncoated: photocatalytically uncoated film; Coated: photocatalytic-coated film. ^c^ Antiviral activity was calculated according to ISO 18071, a guideline of the International Organization for Standardization (see Section 2.5. Criteria of Antiviral Activity of Photocatalytic Material and Effect of Visible Light Irradiation in Materials and methods).

**Table 2:** Inactivation of bovine coronavirus on photocatalytic uncoated and coated films harvested every 1 h with or without 1000 lux light irradiation for 4 h.

Time of Irradiation (h)	Illuminance Values of Visible Light Irradiation (lux)	Virus Titers after Visible Light Irradiation ^a^		Antiviral Activity Values ^c^		
		Uncoated ^b^	Coated ^b^	V_L_	V_D_	_Δ_V
0	0	4.7 (±0.2)	N.T.			
1	0	4.7 (±0.2)	4.0 (±0.3)		0.7	0.2
2	0	4.7 (±0.0)	3.6 (±0.9)		1.1	0.6
3	0	4.5 (±0.2)	3.3 (±0.2)		1.2	0.8
4	0	4.6 (±0.4)	3.1 (±0.4)		1.5	0.7
0	0	N.T.	N.T.			
1	1000	4.5 (±0.0)	3.6 (±0.3)	0.9		
2	1000	4.9 (±0.2)	3.2 (±0.4)	1.7		
3	1000	4.4 (±0.3)	2.4 (±0.2)	2.0		
4	1000	4.5 (±0.2)	2.3 (±0.6)	2.2		

N.T.: not tested; ^a^ Virus titer is shown in log TCID_50_ (tissue culture infective dose 50)/0.1 mL. The virus titer represents the mean (±standard deviation) of three independent experiments. ^b^ Uncoated: photocatalytically uncoated film; Coated: photocatalytic-coated film. ^c^ Antiviral activity was calculated according to ISO 18071, a guideline of the International Organization for Standardization (see Section 2.5. Criteria of Antiviral Activity of Photocatalytic Material and Effect of Visible Light Irradiation in Materials and methods).
